# Bipolar Switching Properties of the Transparent Indium Tin Oxide Thin Film Resistance Random Access Memories

**DOI:** 10.3390/nano13040688

**Published:** 2023-02-10

**Authors:** Kai-Huang Chen, Chien-Min Cheng, Mei-Li Chen, Yi-Yun Pan

**Affiliations:** 1 Graduate Institute of Electronic Engineering, Cheng Shiu University, Kaohsiung 83347, Taiwan; 2 Department of Electronic Engineering, Southern Taiwan University of Science and Technology, Tainan 71005, Taiwan; 3 Department of Electro-Optical Engineering, Southern Taiwan University of Science and Technology, Tainan 71005, Taiwan

**Keywords:** nonvolatile, bipolar switching, RF sputtering, resistance random access memory

## Abstract

In this study, the bipolar switching properties and electrical conduction behaviors of the ITO thin films RRAM devices were investigated. For the transparent RRAM devices structure, indium tin oxide thin films were deposited by using the RF magnetron sputtering method on the ITO/glass substrate. For the ITO/ITO_X_/ITO/glass (MIM) structure, an indium tin oxide thin film top electrode was prepared to form the transparent RRAM devices. From the experimental results, the 10^2^ On/Off memory ratio and bipolar switching cycling properties for set/reset stable states were found and discussed. All transparent RRAM devices exhibited the obvious memory window and low set voltage for the switching times of 120 cycles. The electrical transport mechanisms were dominated by the ohmic contact and space charge limit conduction (SCLC) models for set and reset states. Finally, the transmittances properties of the transparent ITO/ITO_X_/ITO RRAM devices for the different oxygen growth procedures were about 90% according to the UV–Vis spectrophotometer for the visible wavelength range.

## 1. Introduction

According to the dimensions scaled down trend of different traditional memory devise for flash memory, dynamic random access memory (DRAM), and various nonvolatile memory, the reveal charge loss and limiting physical properties were considered and investigated [1,2]. Many advantages of the various nonvolatile memory devices, such as non-volatility, high operation speed of 10 ns, multi-state possibility, the simple capacitor structure of 1–2 nm, small size of 1–2 nm, high packing density of 6F^2^, and low power consumption of 1.5 V properties, were also became prospective candidate for the next-generation nonvolatile memory devices was identified [3,4]. Recently, resistance random access memory (RRAM) devices were widely used for applications in portable electronic systems, such as iPads, cell phones, digital cameras, and flash storage devices. The resistance switching properties were found and discussed from the *I*-*V* characteristics of various thin films RRAM devices between the low resistance state (LRS) and high resistance state (HRS). Many thin film materials for RRAM devices were widely discovered, such as the chalcogenide material, the metal oxide-base, the carbide materials, and amorphous silicon materials, which were used for the RRAM’s applications [5,6,7,8,9,10,11,12].

Seo et al. [13] reported the transparent resistive random access memory (T-RRAM) devices using the ZnO-based material and the electrode of indium tin oxide (ITO) film. In addition, the ITO/ZnO/ITO RRAM devise structure exhibited unipolar switching behavior. In the future, RRAM devices will appear in bionic and high-speed logic operations applications. The T-RRAM device also realized the memory array architecture through the cross array format, memory computing technology of low-power consumption, and developments in the application potential in AI technologies [13]. According to a previous RRAM’s materials-related study, indium tin oxide (ITO) was an important candidate for the transparent electrode materials for its high thermal stability properties, large band gaps, good transmittances, and electrical conduction properties [14,15]. For the motivation of the transparent RRAM device’s fabrication in this study, the ITO_X_ material was chosen because of the low-cost single processing and the continuous deposition time of the same one RF sputtering vacuum system. In addition, the high transparent properties, high conductivity, electrical resistive switching behavior, bipolar switching properties, electrical conduction transport mechanisms, and On/Off memory ratio of the transparent ITO/ITO_X_/ITO RRAM devices were important challenge for application in future memory devices. The transmittance of the ITO/ITO_X_/ITO RRAM devices will be discussed and affected by the different oxygen concentration deposited parameters.

## 2. Experimental Procedures

**Figure 1:**
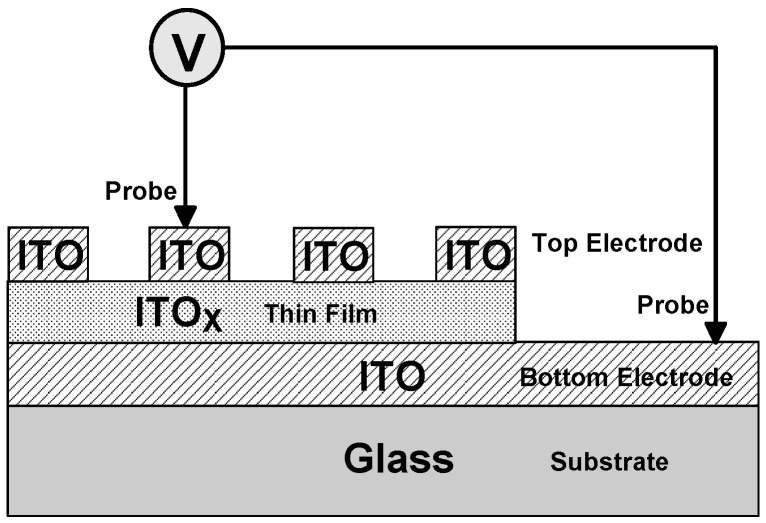
The MIM structure of the transparent ITO/ITO_X_/ITO RRAM device.

For ITO_X_/ITO RRAM structure, the ITO_X_ thin films were deposited on the ITO/glass substrate by RF power of 50 W, chamber pressure of 20 mTorr, and sputtering time of 10 min. The XRD patterns of the ITO_X_ thin films were recorded to determine the crystalline phase of the X-ray diffraction (XRD) in the 2θ degree of 20–60°. The surface micro-structure and cross-section morphology were observed by scanning electron microscope (SEM). Using the RF magnetron sputtering method, the indium tin oxide top electrode (diameter = 0.1 cm) array deposited (with 100% Ar, ITO target) for the transparent metal-insulator-metal (MIM) (ITO/ITO_X_/ITO) RRAM structure was shown in Figure 1. The current versus applied voltage (*I-V*) curves switching characteristics of the ITO/ITO_X_/ITO RRAM structure were measured by the semiconductor parameter analyzer (HP4156C). The electrical transport conduction mechanisms of set/reset state were discussed and investigated. In addition, the transmittances of the ITO/ITO_X_/ITO RRAM were measured using the UV–Vis spectrophotometer for the visible wavelength range of 400–800 nm. In this study, the transparent ITO/ITOx/ITO/glass transparent RRAM devices for 10 samples were measured.

## 3. Results and Discussion

**Figure 2:**
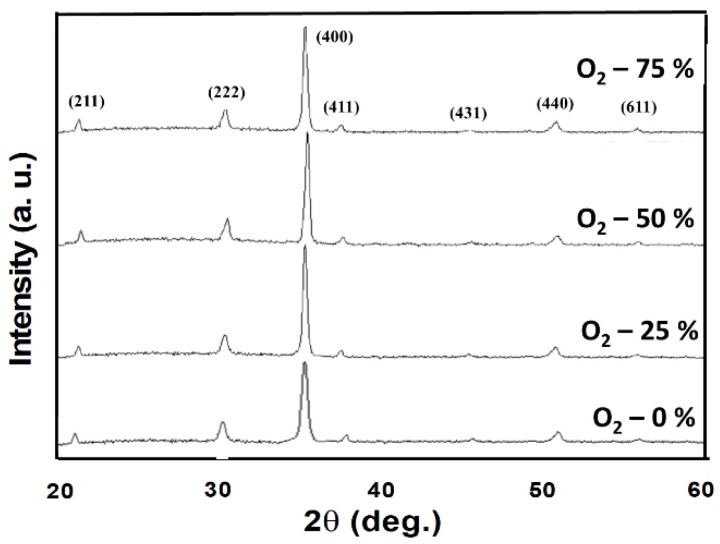
XRD patterns of the as-deposited ITO_X_ thin films under different oxygen growth procedure parameters.

**Figure 3:**
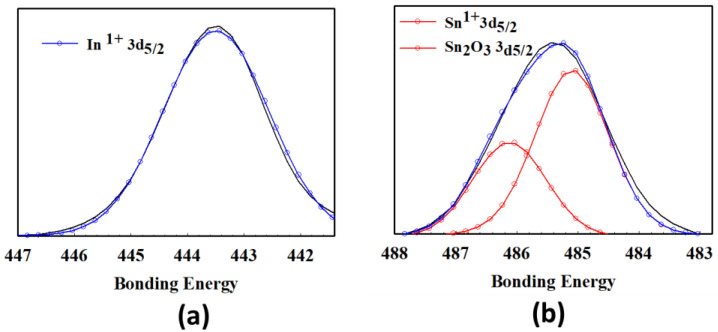
The XPS results of the ITO_X_ thin films for (**a**) In^1+^ 3d_5/2_, and (**b**) Sn^1+^ 3d_5/2_, Sn_2_O_3_ 3d_5/2_ peaks.

Figure 2 depicted the XRD patterns of the as-deposited ITO_X_ thin films prepared by RF power of 50 W for different oxygen growth procedure parameters. All ITO_X_ thin films were exhibited the mainly preferred (400) phrase orientation for 2θ degree of 35°. In addition, the (211), (222), (400), (411), (431), and (440) peaks of thin films were observed in XRD patterns in previous studies [16,17,18]. The (211), (200), and (440) peaks corresponding to the ITO_X_ thin films were also observed in the XRD pattern. The XRD results demonstrated that the (400) peaks of the ITO_X_ phases were obtained for different oxygen growth procedure parameters. In XRD patterns, the sharp and strong (400) phrase preferred peak of ITO_X_ thin film was found and chosen in the following experimental procedure. Figure 3 shows the cross-sectional SEM image of the RRAM device’s structure for different oxygen growth procedure parameters. The layer structures and evident interface of ITO_X_, ITO, and glass were observed and found. In addition, the thickness of as-deposited ITO_X_ thin films was calculated to 228 nm.

Figure 3 presented the XPS measurement of the transparent ITO_X_ thin films for (a) In^1+^ 3d_5/2_, and (b) Sn^1+^ 3d_5/2_, Sn_2_O_3_ 3d_5/2_ peaks. In Figure 3, the In^1+^ 3d_5/2_ and Sn^1+^ 3d_5/2_ bonding energy of the transparent ITO_X_ thin films was 443 eV and 485.3 eV, respectively. In Table 1, the mole fraction ratio of the transparent as-deposited and RTA–ITO_X_ thin films for Sn, In, and O elements was observed from the XPS results. The oxygen mole fraction of as-deposited ITO_X_ thin films for 50% oxygen growth procedure parameters was about 47.15% in Table 2. The oxygen mole fraction of RTA–ITO_X_ thin films was increased to 76.98% in Table 1. This result proved the oxygen mole fraction was increased by RTA processing using oxygen gas treatment. However, the ITOx thin film for 50% oxygen growth procedure parameters exhibited low oxygen content of thin films. As shown in Figure 2, the sharp (400) phrase preferred peak of ITO_X_ thin film for 50% oxygen growth procedure parameters was chosen in the following experimental procedure. In addition, the ITO_X_ thin film for different oxygen growth procedure parameters which exhibited the high memory ratio was also shown in *I-V* curves of RRAM devices.

**Figure 4:**
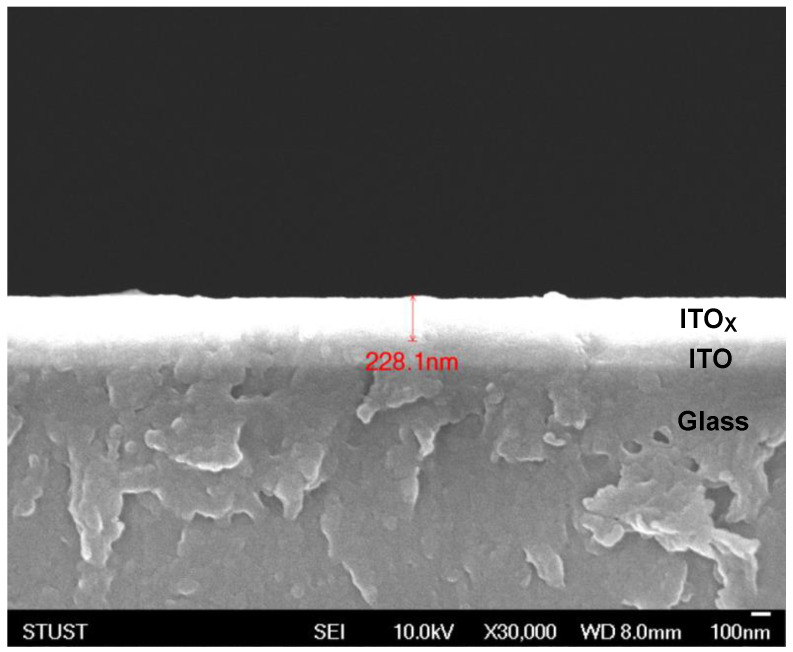
The SEM cross-section image of the as-deposited ITO_X_ thin film for 50% different oxygen growth procedure parameters.

**Figure 5:**
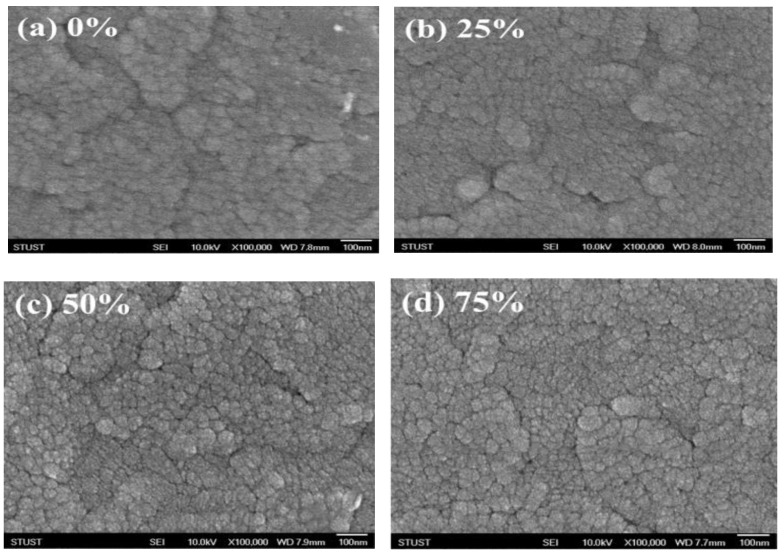
The surface micro-structure images of ITO_X_ thin films prepared by different oxygen growth procedure parameters for (**a**) 0%, (**b**) 25%, (**c**) 50%, and (**d**) 75%.

Figure 4 depicts the surface micro-structure morphologies of ITO_X_ thin films at different oxygen growth procedure parameters. More non-uniform grains and rather bumpy surfaces were found in 0% and 25% oxygen growth procedure parameters. As shown in Figure 5c,d, the denser surfaces of thin films for 50% and 75% oxygen growth procedure parameters was increased. In addition, the oxygen vacancies were the main factor for the electrical current transport paths under the different oxygen sputtering growth procedure parameters. The electrical transport conduction paths affected by the variation in oxygen vacancies of ITO_X_ thin films for the 50% oxygen growth procedure parameters were improved in *I*-*V* curves switching behaviors.

**Figure 6:**
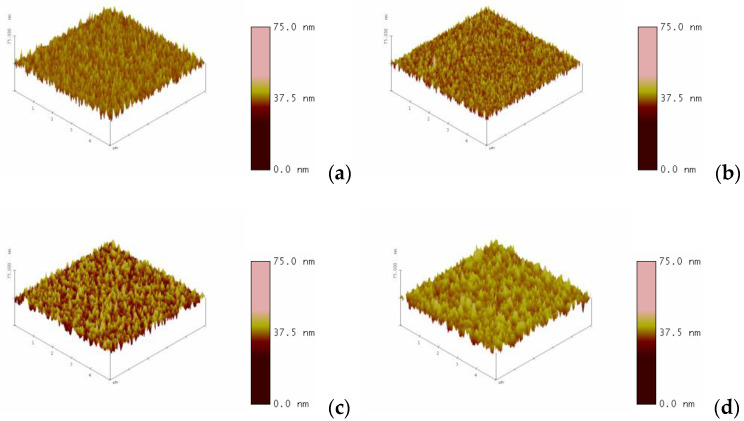
The surface micro-structure (AFM) images of the ITO_X_ thin films prepared by different oxygen growth procedure parameters for (**a**) 0%(**b**) 25% (**c**) 50%, and (**d**) 75%.

Figure 6 presents the surface micro-structure (AFM) images of the ITO_X_ thin films prepared by different oxygen growth procedure parameters. For the inhomogeneity of layer thickness, the surface roughness of the ITOx thin films was also observed and discussed. The surface roughness of the ITO_X_ thin films for different oxygen growth procedure parameters was about 3.434, 2.371, 3.821, and 3.145 nm, respectively. For 50% oxygen growth procedure parameters, the large roughness surface and the non-flat depth images of the inhomogeneity ITOx layer thickness were caused by excess oxygen gas growth procedure parameters and re-sputtering treatment.

**Figure 7:**
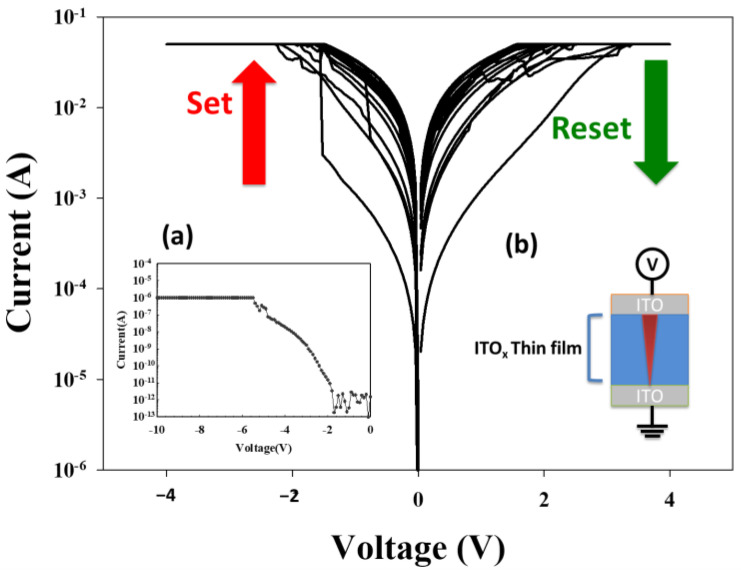
The *I-V* curves of ITO/ITO_X_/ITO RRAM for (**a**) initial electrical forming process, and (**b**) electrical filament mechanism model on the MIM structure.

The typical *I–V* curves of the ITO_X_ thin films RRAM devices are shown in Figure 7. The electrical current compliance is 5 mA in Figure 7a. The MIM capacitor structure of the ITO/ITO_X_/ITO RRAM devices is shown in Figure 7b. For the initial forming process at a voltage of −10 V, the ITO_X_ thin films from the RRAM devices switch to the low resistance state (called LRS) and the high resistance state (called HRS). To discuss and confirm the stable *I–V* switching behavior, the LRS/HRS states of the ITO_X_ thin films of the RRAM devices were repeated 100 times.

**Figure 8:**
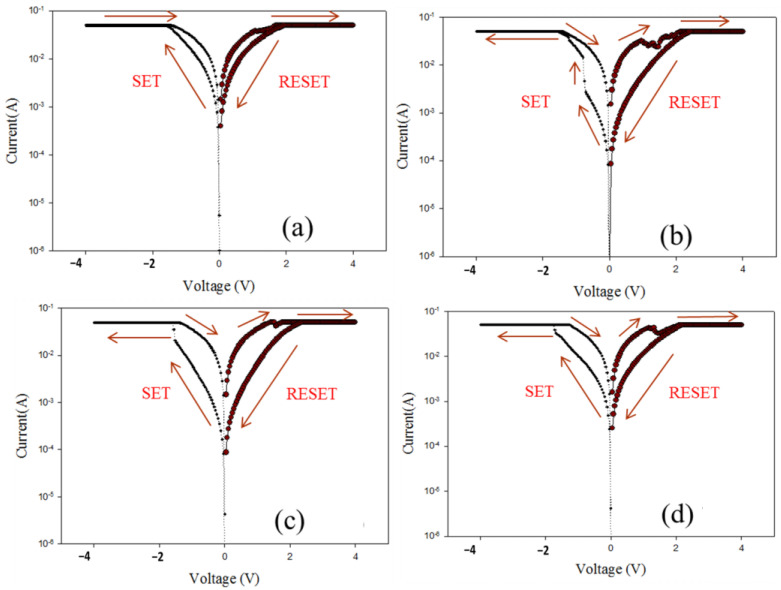
The *I-V* curves of ITO/ITO_X_/ITO RRAM under different oxygen growth procedure parameters for (**a**) 0%, (**b**) 25%, (**c**) 50%, and (**d**) 75%.

For the RF sputtering process parameters, the different defects and oxygen vacancies of as-deposited ITO_X_ thin films were filled and compensated for different oxygen growth procedure parameters. Figure 8 shows the *I-V* characteristics of ITO/ITO_X_/ITO RRAM for different oxygen growth procedure parameters. All RRAM devices exhibited the bipolar switching behavior in the *I-V* curves result. The On/Off memory ratio of ITO/ITO_X_/ITO RRAM devices for 50% oxygen growth procedure parameters was also observed in the *I-V* curves.

Some studies reported that the oxygen vacancies effect played an important role in the resistive switching properties of the RRAM devices [19,20,21]. In the initial metallic filament model, the transport formation path and rupture effect of the electrical conducting filaments were the main electrical switching mechanisms. The electrical switching transport mechanism of the ITOx thin film was correlated to oxygen ions or to vacancies between of the electrode and the insulator thin film. As shown in Figure 8a–d, the set voltage of RRAM devices gradually increases from −0.7 V (25% O_2_), −1.7 V (50% O_2_), and −1.8 V (75% O_2_). In addition, the obvious *I-V* switching characteristic of the RRAM devices for 0% oxygen growth procedure parameters was not observed.

**Figure 9:**
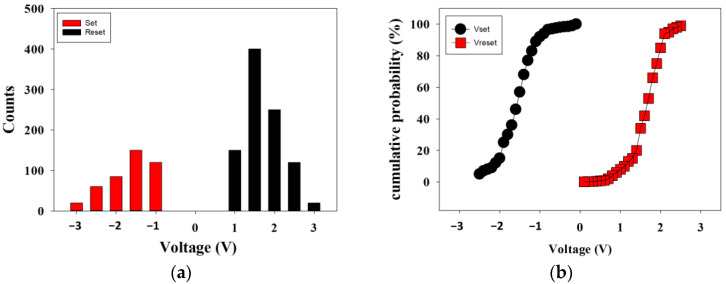
(**a**) The counts distribution results, and (**b**) the statistical results of cumulative probability for the resistive switching properties of the RRAM devices in set and reset (400 times).

Figure 9a,b presents the statistical results of distribution of the set/reset voltages measured from the RRAM devices for 400 times measured, respectively. In Figure 9a, the counts distribution results, the set and reset voltage value was found for the range of applied voltage of −3 to 3 V. In addition, the statistical results of cumulative probability for the resistive switching properties of the RRAM devices in set and reset are also observed in Figure 9b.

Many previous studies have suggested ways to prove the electrical transport conductive behaviors of the RRAM devices: the modification of the Schottky mechanism for the trapped charge carrier model, the conductive filamentary path model, the migration of oxygen vacancies model, and the carriers tunneling model [22,23,24,25]. In addition, many studies attempted to elucidate the *I-V* resistive switching properties of RRAM devices.

**Figure 10:**
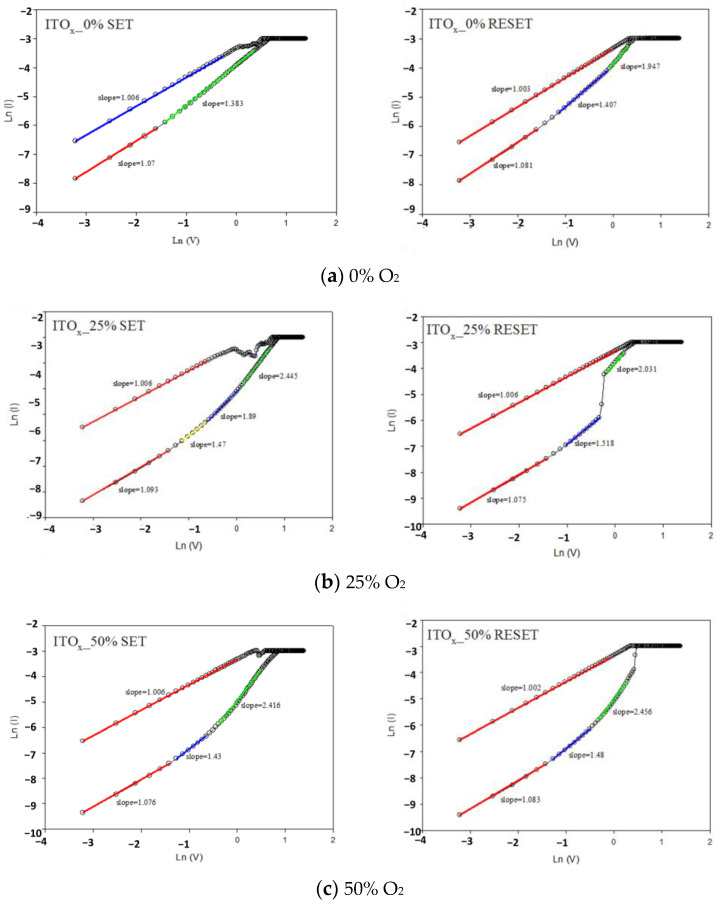

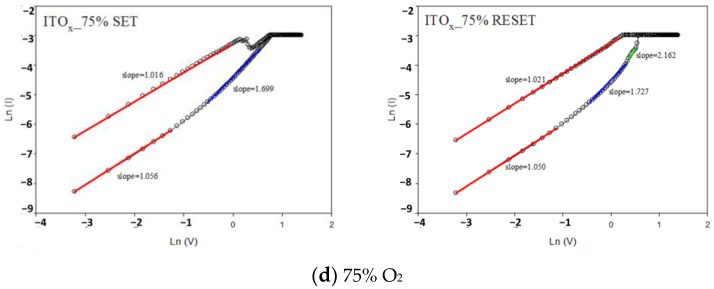
The Ln (I)-Ln (V) plot of ITO/ITO_X_/ITO RRAM for different oxygen growth parameters (RF power of 50 W) for (**a**) 0%(**b**) 25%(**c**) 50%, and (**d**) 75%.

Figure 10 depicts the *I-V* curves characteristics (Ln (I) as the vertical axis and Ln (V) as the horizontal axis) of the ITO/ITO_X_/ITO RRAM devices under 0, 25, 50, and 75% different oxygen growth procedure parameters. According to experimental results, the electrical current conduction mechanism was ohmic conduction (external electric field dominated, slope ≈ 1) for low voltages. As high voltages were applied, the defects filled by the carriers of electrical conduction mechanisms were caused by the space charge limit conduction (SCLC, slope ≈ 2). The electrical conduction mechanisms exhibited both ohmic conduction and SCLC mechanisms (slope = 1~2.5). The electrical current density of the ohmic conduction and SCLC was written as [26]:

(1) $$J=\mathrm{nq}\mu E\exp(\frac{-\Delta E_{ac}}{kT})$$

(2) $$J=\frac{9\varepsilon_{i}\mu V^{2}}{8d^{3}}.$$

In Equation (1), J is the electrical current density, E is the electric field, *μ* is the mobility of electrons, ΔE_ac_ is the activation energy of electrons, T is the absolute temperature, k is the Boltzmann constant, n is the electron concentration, d is the thickness of the insulator layer, *ε*_i_ is the dynamic permittivity of the insulator layer, and V is the applied voltage.

**Figure 11:**
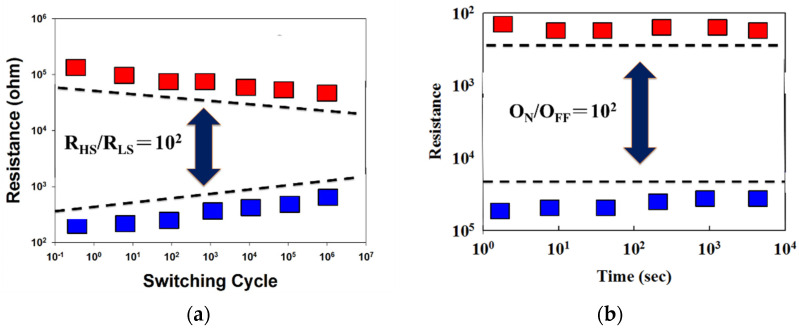
Switching endurance of the transparent ITO/ITO_X_/ITO RRAM for (**a**) retention properties, and (**b**) endurance properties (measured at room temperature).

Figure 11 presents the switching cycling versus resistance value curves, measured by using the type of the retention and endurance measurement properties. In Figure 11a, the retention properties of the ITO/ITO_X_/ITO thin film RRAM devices were measured to investigate their reliability for applications in non-volatile memory RRAM devices. Figure 11b demonstrates that no significant changes in the O_N_/O_FF_ ratio switching resistance cycling versus testing time curves in ITO/ITO_X_/ITO thin film RRAM devices were found for more than 10^2^ s in the extrapolation calculation anticipation measured.

**Figure 12:**
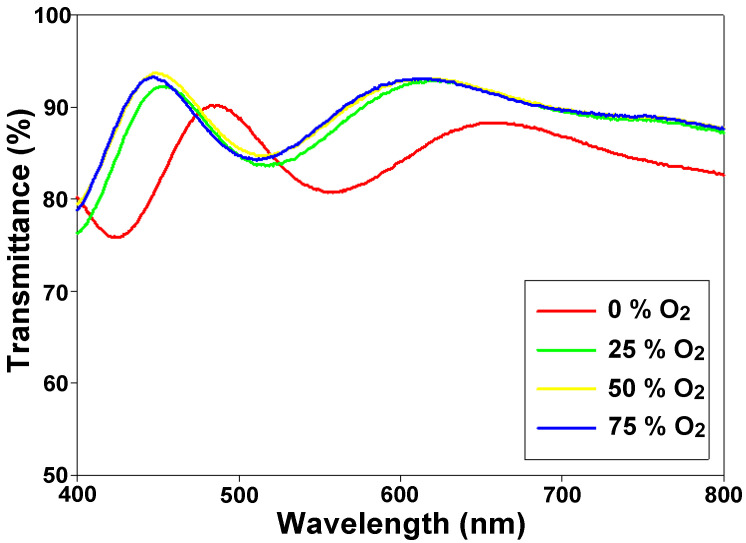
The transmittance of ITO/ITO_X_/ITO RRAM for the visible wavelength range of 400–800 nm.

**Figure 13:**
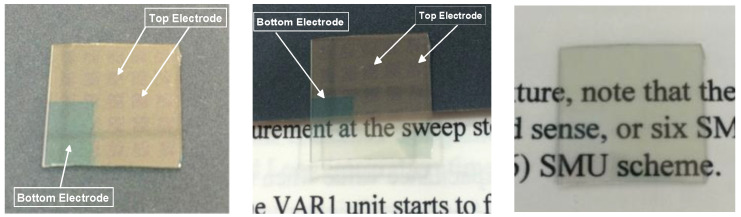
Optical images of the transparent ITO/ITO_X_/ITO RRAM device.

For the visible wavelength range from 400 to 800 nm, the transmittance properties of ITO/ITO_X_/ITO RRAM devices were measured in Figure 12. For the 0% oxygen growth procedure parameters, the transmittance efficiency was about 85% for all visible wavelength ranges. The optimal transmittance efficiency of 93% for 50% oxygen growth procedure parameters was observed. Additionally, the transparency of ITO/ITO_X_/ITO RRAM devices was improved to 90%. The transmittance efficiency and resistivity value of the as-deposited ITO_x_ thin film for RTA treatment were about 94% and 5 × 10^−1^ (Ω-cm). Finally, the good transparency efficiency of the ITO/ITO_X_/ITO RRAM as observed by optical images is shown in Figure 13.

**Figure 14:**
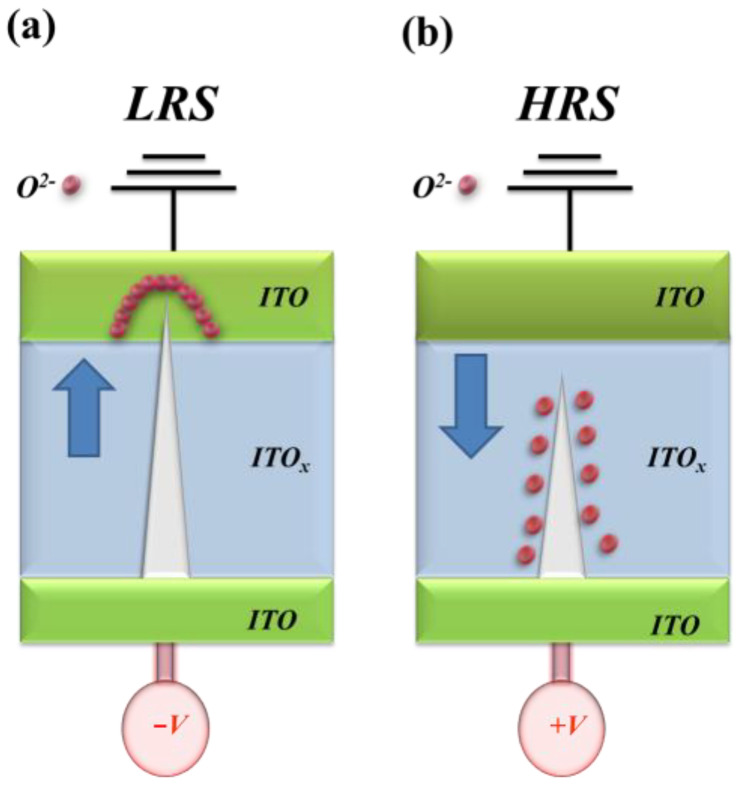
The initial metallic filament path model of the transparent ITO/ITO_X_/ITO RRAM device for (**a**) LRS (set), and (**b**) HRS (reset) state.

Figure 14 presents the initial electrical metallic filament forming physical model of the ITO/ITO_X_/ITO thin film RRAM devices for LRS/HRS state. In Figure 14a, the initial electrical metallic filament path model for the ITO/ITO_X_/ITO thin film RRAM devices for negative bias in the set state. As shown in fig. 14a, the oval pattern of the depletion region formed by the oxygen ions and vacancies in the ITO top-electrode of the transparent ITO/ITO_X_/ITO thin films RRAM devices for LRS was gradually accumulated. In addition, the electrical metallic path tip passed through the oval depletion region in the ITO electrode using the application of continuous high negative voltage. The thin electrical metal metallic filament for the continuous oxidation reaction from oxygen atoms and vacancies with the application of high positive voltage was shown in Figure 14b. The electrical metal metallic filament is continuously sharp for the oxygen atoms and vacancies in the ITO bottom electrode. Finally, the symmetry bipolar switching *I-V* curves properties of the transparent ITO/ITO_X_/ITO RRAM devices were also proved and verified by the physical forming model of the top/bottom ITO electrode [19,20,21,22,23,24,25].

## 4. Conclusions

The electrical bipolar resistance switching properties of ITO_X_ thin films RRAM devices were well investigated and discussed. The high-performance of simple capacitor structure, high transmittance efficiency, and the stable switching cycle of the ITO/ITO_X_/ITO structure RRAM devices for applications in nonvolatile memory devices. The RRAM devices all exhibited the bipolar switching behavior and memory window of 10^2^ on/off ratio. In addition, the memory reliability for switching endurance and retention testing was also found. The electrical conduction mechanisms for LRS/HRS states were exhibited ohmic and SCLC mechanisms for SET/RESET states. The transparency properties of ITO/ITO_X_/ITO RRAM devices were improved from 80% (0% O_2_) to about 90% (>25% O_2_). The low power operation and high uniformity properties of the transparent ITO/ITO_X_/ITO RRAM devices will be a candidate for high density and low power consumption electronic devices.

## Figures and Tables

**Table 1:** The mole fraction ratio of the ITO_X_ thin films from the XPS results.

Mole fraction	Sn	In	O
ITO	5.08%	47.76%	47.15%
RTA-ITO	4.7%	18.32%	76.98%

**Table 2:** The oxygen gas present of the growth procedure and oxygen fraction of as-deposited thin film from the XPS results.

Growth Procedure Argon: Oxygen Gas (%)	Thin Film Oxygen Mole Fraction (%)
0%	47.15%
25%	52.1%
50%	55.2%
75%	57.4%

## Data Availability

Availability Statements are available in section “MDPI Research Data Policies” at https://www.mdpi.com/ethics (accessed on 29 December 2022).
